# Resuscitative endovascular balloon occlusion of the aorta for uncontrollable nonvariceal upper gastrointestinal bleeding

**DOI:** 10.1186/s13017-016-0076-3

**Published:** 2016-05-20

**Authors:** Hidefumi Sano, Junya Tsurukiri, Akira Hoshiai, Taishi Oomura, Yosuke Tanaka, Shoichi Ohta

**Affiliations:** Emergency and Critical Care Medicine, Tokyo Medical University Hachioji Medical Center, 1163 Tatemachi, Hachioji, Tokyo 193-0998 Japan; Emergency and Disaster Medicine, Tokyo Medical University, 6-7-1 Nishi-shinjuku, Shinjuku, Tokyo, 160-0023 Japan

## Abstract

**Background:**

Although resuscitative endovascular balloon occlusion of the aorta (REBOA) in various clinical settings was found to successfully elevate central blood pressure in hemorrhagic shock, this intervention is associated with high mortality and may represent a last-ditch option for trauma patients. We conducted a retrospective study of patients with nonvariceal upper gastrointestinal bleeding (UGIB) who underwent REBOA to identify the effectiveness of REBOA and reviewed published literatures.

**Methods:**

REBOA were performed by trained acute care physicians in the emergency room and intensive care unit. The deployment of balloon catheters was positioned using ultrasonography guidance. Collected data included clinical characteristics, hemorrhagic severity, blood cultures, metabolic values, blood transfusions, REBOA-related complications and mortality. A literature search using PUBMED to include “aortic occlusion” and “gastrointestinal bleeding” was conducted.

**Results:**

REBOA was attempted in eight patients among 140 patients with UGIB and median age was 66 years. Systolic blood pressure significantly increased after REBOA (66 ± 20 vs. 117 ± 45 mmHg, *p* < 0.01) and the total occlusion time of REBOA was 80 ± 48 min. Strong positive correlations were found between total occlusion time of REBOA and lactate concentration (Spearman’s *r*=0.77), clinical Rockwall score (Spearman’s *r*=0.80), and age (Spearman’s *r*=0.88), respectively.

**Conclusion:**

REBOA can be performed with a high degree of technical success and is effective at improving hemodynamic in patients with UGIB. Correlations between total occlusion time and high lactate levels, clinical Rockall score, and age may be important for successful use of REBOA.

## Background

Uncontrollable hemorrhage is a main cause of death in patients with hemorrhagic shock admitted to the emergency department (ED) or intensive care unit (ICU), and trauma and nonvariceal upper gastrointestinal bleeding (UGIB) are the most common causes of massive hemorrhage in acute care setting [1, 2]. Although main aim of resuscitation is to stop the source of hemorrhage and restore hemodynamics, persistent hemorrhage can be rapidly fatal. The options for impending cardiac collapse are resuscitative thoracotomy and aortic clamping immediately performed in such cases [3].

A recent systematic review of REBOA in various clinical settings was found to successfully elevate central blood pressure in hemorrhagic shock [4]. Although, REBOA is increasingly used as an alternative to resuscitative thoracotomy, a recent report suggested that REBOA was associated with increased mortality and may represent a last-ditch option for trauma patients with hemodynamic instability in Japan [5]. However, there are no satisfactory reports regarding the effectiveness of REBOA among patients with UGIB. Therefore, we conducted a retrospective study of patients with UGIB who underwent REBOA at a single emergency center to evaluate the effectiveness of REBOA. In addition, we reviewed the published literature to provide a summary of the experience data.

## Methods

### Patients

The ethics committee of Tokyo Medical University Hachioji Medical Center approved the design of this retrospective study. UGIB patients with suspected hemorrhagic shock who subsequently underwent REBOA in the ER or who were admitted to our intensive care unit (ICU) and subsequently developed hemorrhagic shock and underwent REBOA in the ICU were enrolled in this study between September 2011 and April 2015. Patients with a systolic blood pressure (SBP) <90 mmHg or a shock index (SI; ratio of heart rate to SBP) ≥1.0 were considered to be in shock. We excluded patients aged <15 years and those who had cardiac arrest on admission or were diagnosed with any terminal disease during the study period.

### Intervention

Patients with hemorrhagic shock in the transient- and non-response groups were considered to be hemodynamically unstable on the basis of their response to an initial fluid resuscitation with 1 L of Ringer’s lactate. Although it is important to administer blood and blood products as soon as possible for trauma or non-trauma shock patients, the preparation of blood or blood products takes time in Japan, at least in our hospital. Consequently, the empirical transfusion of blood and blood products was initiated as soon as possible. REBOA was initiated by one or two acute care physicians in patients showing hemodynamic instability and an inability to remain normotensive following resuscitation. In our department, one acute care physician (TJ) was trained for ≥1 year as a member of the endovascular team in the Radiology Department of another university hospital, whereas all other acute care physicians in our ER performed REBOA under the guidance of TJ.

**Fig. 1:**
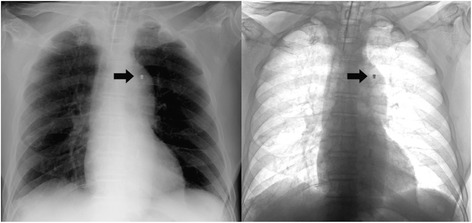
Placement of the balloon is performed in Zone I (Patient 5). The tip of IABO catheter (arrow)

For the REBOA procedure, a 10 Fr. Intra-aortic balloon occlusion (IABO) catheters (BLOCK BALLOON™; Senko Medical Instrument, Tokyo, Japan) or 7 Fr. IABO catheters (RESCUE BALLOON®; Tokai Medical Products, Tokyo, Japan) have been available in our ER and ICU. These are a double lumen balloon catheter with a stainless steel styled. For percutaneous deployment of IABO catheters, all necessary guidewires, sharps and introducers are packaged together in the kit. An acute care physician first inserts 7 Fr. or 10 Fr. sheath into the femoral artery using the Seldinger method. After insertion of the femoral artery sheath, the IABO catheter was placed into the aorta and REBOA was performed. IABO catheter was placed into the aorta, with selection of the aortic zone for occlusion according to the recommendations of Stannard et al. under ultrasonography guidance [6]. Placement of the balloon is normally performed in Zone I (proximal of the aorta, origin of the left subclavian artery to the celiac artery) in patients with suspected UGIB. IABO catheter positioning was performed under ultrasonography guidance before REBOA placement and confirmed by portable chest radiography in ER (Fig. 1) [7].

### Data collection

The following characteristics were noted from the charts and radiographs of the patients with hemodynamic instability: age, sex, vital signs, Acute Physiology and Chronic Health Evaluation (APACHE) II score, hemorrhagic severity, blood cultures, metabolic and coagulation values [pH, lactate concentration, base excess (BE), prothrombin time, and activated partial thrombin time], blood transfusion, REBOA-related complications and mortality. Hemorrhagic severity was evaluated using SI and severity of UGIB was evaluated at the onset using the Glasgow-Blatchford bleeding score (GBS), clinical Rockall score (CRS) and AIMS65 score [8]. In patients admitted to ER or ICU, blood cultures and metabolic and coagulation values were measured at the beginning of resuscitative interventions. Markers of end-organ dysfunction included serum aspartate transaminase (AST), blood urea nitrogen (BUN), creatinine, potassium, sodium, total bilirubin, lactate dehydrogenase (LDH), and creatine kinase (CK) as described in previous reports thoroughly reviewed by two acute care physicians (SH and TJ) [9, 10]. These values were measured within 12 h after definitive hemostasis. The REBOA-related complications included vessel injuries (i.e., aortic dissection, rupture, perforation, pseudo-aneurysm, and arteriovenous fistula), groin hematoma, embolization, air emboli, peripheral ischemia, and organ dysfunction.

### Literature search

A literature search using PUBMED to include “aortic occlusion” and “gastrointestinal bleeding” was conducted. Original articles and case reports published in English language were reviewed, and follow-up references listed were further investigated.

### Statistical analyses

Data from all eligible patients were analyzed. Continuous variables are shown as mean values with standard deviation in text and median and interquartile range in Tables. Between-group differences were statistically assessed using the Mann–Whitney *U* test for continuous variables, paired t test for continuous dependent variables and the Fisher’s exact test for categorical variables. The Spearman correlation coefficient was used to identify correlations between the evaluated parameters. All statistical analyses were performed using Prism version 6.0a statistical software (GraphPad Software, San Diego, CA, USA). Categorical variables were calculated as the ratio (percentage) of the frequency of occurrence. A probability (*p*) value of > 0.05 was considered statistically significant.

## Results

### Demographics and clinical characteristics

REBOA was attempted in eight patients among 140 patients with UGIB. The mean age was 66 ± 16 years, and all of the patients were male. The mean SI, GBS, CRS, AIMS65 score and APACHE II were 1.5 ± 0.6, 16 ± 4, 3 ± 1, 2 ± 2, and 23 ± 7, respectively. The demographics and clinical characteristics of all patients are shown in Table 1. Placement of the IABO catheter failed in one patient aged 79 years with severe aortic calcifications. Definitive hemostasis was endoscopy in 3 cases, anigo-embolization (AE) in 2 cases, and AE after failed endoscopy in 3 cases, respectively. The total occlusion time of REBOA was 80 ± 48 min in this study. The mean volume of packed red blood cells, fresh frozen plasma and Ringer’s lactate administered during the resuscitation were 2000 ± 949 mL, 1440 ± 733 mL, and 4000 ± 2363 mL, respectively. The mortality rates within 24 h and 30 days were 15 % each. No REBOA-related complications were encountered.

**Table 1:** Clinical characteristics of the patients

Variables	Patient 1	Patient 2	Patient 3	Patient 4	Patient 5	Patient 6	Patient 7	Patient 8
Age (y)	69	36	83	64	78	69	50	79
Sex	male	male	male	male	male	male	male	male
Body temperature (°C)	35.5	36.2	36.8	37.3	36.0	37.0	36.1	35.7
Hemodynamics								
Shock index	2.3	0.8	2.1	2	0.8	1.7	1.1	1.3
SBP pre-REBOA (mmHg)	60	90	96	54	41	63	61	–
SBP post REBOA (mmHg)	97	111	206	74	82	112	140	–
ΔSBP (mmHg)	37	21	110	20	41	49	79	–
APACHE II	22	7	26	31	22	26	21	30
Severity of upper gastrointestinal bleeding								
Glasgow Blatchford bleeding score	17	7	19	11	14	13	12	17
Clinical Rockall score	3	3	4	3	5	3	2	3
AIM 65 score	3	0	4	3	2	5	0	2
Initial blood values								
White blood cell count (/μL)	12900	13600	24000	17300	9120	10600	11500	7170
Hemoglobin (g/dL)	5.5	14.8	11.2	7.3	5	5.3	8.8	6.1
Hematocrit (%)	16	44	31	21	15	16	26	19
Platelet connt (×10^4^/μL)	11.9	34.6	9.3	36.8	17.3	12.2	36	22.9
BUN (mg/dL)	18.7	21.1	62.1	8.2	44.1	13.9	46.9	32.0
Lactate (mmol/L)	11.1	1.3	9.6	5.6	15.8	6.6	63	15.0
pH	7.25	7.36	7.38	6.94	7.35	7.24	7.30	6.96
Base excess (mmol/L)	0.1	–2.5	−11.5	−14.5	−14.6	–9.3	–9.4	–21.8
Prothrombin time (%)	51	113	64	62	83	51	105	62
APTT (sec)	64.5	30.1	35.3	240	64.3	123.9	33.4	–
Diagnosis	Gastric ulcer	Gastric ulcer	Duodenum ulcer	Anastomotic bleeding	Duodenum ulcer	Left gastric artery aneurysm	Duodenum ulcer	Esophageal cancer
Definitive hemostatic control	endoscopy	endoscopy	endoscopy	AE	AE (failed endoscopy)	AE (failed endoscopy)	AE (failed endoscopy)	AE
CPA during procedures	no	no	no	yes	no	yes	no	no
Blood and blood product transfusion within 24 h (mL)								
PRBC	1400	280	1960	2520	1960	2800	3080	1680
FFP	1200	0	1200	1680	2160	1920	1920	720
Re-bleeding	no	no	no	no	yes	yes	no	no
REBOA								
Total occlusion time (min)	57	20	140	54	145	95	50	Failure
REBOA-related complication	none	none	none	none	none	none	none	none
Outcome within 30 days	Alive	Alive	Alive	Alive	Died < 24 h	Alive	Alive	Died < 24 h

*SBP* systolic blood pressure, *REBOA* resuscitative endovascular balloon occlusion of the aorta, *APACHE* acute physiology and chronic health evaluation, *BUN* blood urea nitrogen. *APTT* activated partial thrombin time, *AE* angioembolization, *CPA* cardiopulmonary arrest, *PRBC* packed red blood cells, and *FFP* fresh-frozen plasma

### Changes in acute care management with REBOA

Systolic blood pressure was significantly higher after initiating of REBOA (66 ± 20 vs. 117 ± 45 mmHg, *p* < 0.01). Heart rate, lactate concentration, and BE were not significantly different between before and after REBOA. Initial serum concentration of AST, BUN, creatinine, CK, potassium, sodium, total bilirubin, white blood cell counts, and C-reactive response were not significantly different compared with those after completion of hemostasis. The serum concentration of LDH following REBOA was significantly higher than that before REBOA (227 ± 154 vs. 595 ± 406 IU/L, *p*=0.04) (Table 2).

**Table 2:** Comparison of the vital indicators between before and after REBOA

Vital indicators, median (IQR)	Before REBOA (*n*=7)	After REBOA (*n*=7)
Systolic blood pressure (mmHg)	61 (57–77)	111 (90–126)*
Heart rate (beat/min)	126 (112–131)	123 (113–127)
White blood cells (/μL)	12100 (11050–14250)	16600 (13950–17900)
Hemoglobin (g/dL)	7.3 (5.4–10)	9.7 (8.7–10.6)
Platelet (×10^4^/μL)	17.3 (12.1–35.3)	10.3 (7.2–13.6)
Prothrombin time (%)	64 (57–94)	56 (50–70)
Lactate (mmol/L)	3.2 (2.9–6.0)	5.4 (4.3–7.0)
Base excess (mmol/L)	−11.5 (−14.6 - –8.7)	−3.1 (−4.8 - –1.4)
Blood urea nitrogen (mg/dL)	21.1 (16.3–45.5)	30.8 (16.6–38.8)
Creatinine (mg/dL)	0.97 (0.90–1.03)	1.14 (0.83–1.29)
Aspartate transaminase (U/L)	14 (11.5–43.5)	101 (64.5–608)
Total bilirubin (mg/dL)	0.40 (0.35–0.6)	0.60 (0.45–0.80)
Potassium (mmol/L)	4.0 (3.8–4.1)	3.5 (3.5–4.2)
Sodium (mmol/L)	138 (135–139)	137 (136–141)
Creatine kinase (U/L)	56 (47–80)	263 (109–620)
Alkaline phosphatase (U/L)	191 (153–197)	137 (104–249)
Lactate dehydrogenase (U/L)	158 (123–285)	411 (328–862)*
C reactive protein (mg/dL)	1.61 (0.55–2.64)	1.07 (0.70–3.55)

*IQR* interquartile range, *REBOA*, resuscitative endovascular balloon occlusion of the aorta; * = *p* < 0.05 vs. Before REBOA

### Correlations between total occlusion time and vital indicators

**Fig. 2:**
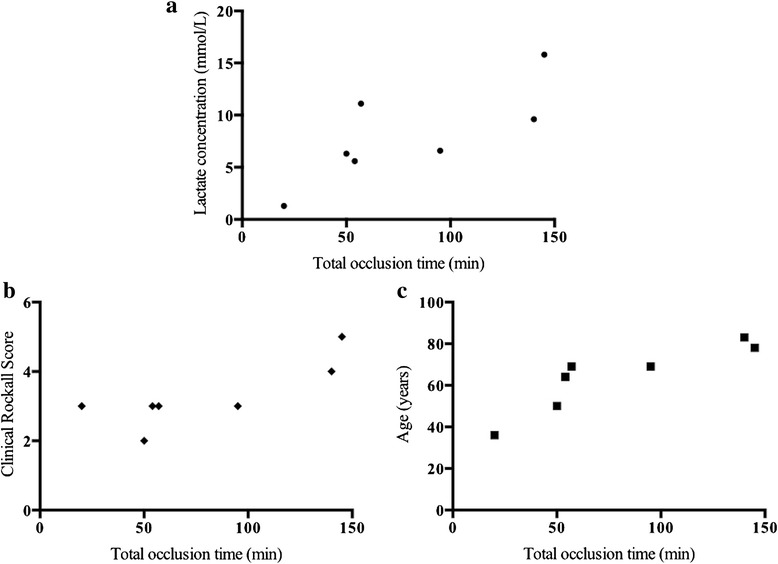
Significantly positive correlation between the total occlusion time of the intra-aortic balloon occlusion catheter and lactate concentration, clinical Rockall score and age. **(a)** Lactate concentration, Spearman’s *r*=0.77; (**b**) clinical Rockall score, Spearman’s *r*=0.80; and (**c**) age, Spearman’s *r*=0.88

Strong positive correlations were found between total occlusion time and lactate concentration (Spearman’s *r*=0.77, *p*=0.04), CRS (Spearman’s *r*=0.80, *p*=0.03), and age (Spearman’s *r*=0.88, *p* < 0.01), respectively (Fig. 2).

## Discussion

REBOA is an adjunct procedure designed to sustain the circulation until definitive hemostasis is obtained.

Upon review of the existing literature, it was evident that there are only a very limited number of publications related to REBOA treatment for UGIB, and only four case references were retrieved. We identified 4 published reports involving 4 patients (1 patient per 1 report) with hemodynamic instability caused by UIGB. Among them, 2 patients underwent REBOA for duodenum ulcers, one child for aorta-esophageal fistula, and one for unknown UGIB [11–14]. Table 3 showed the clinical characteristics. Placement of the balloon was in Zone I without fluoroscopy and hemodynamics have improved in all cases. There was no REBOA-related complication and the mortality rate was 25 %. To the best of our knowledge, the present study represents the first retrospective study to evaluate the utility of REBOA among patients with UGBI.

**Table 3:** Literature review

	Author	Year of publication	Study type	Number of patient	Age (years)	Sex	Diagnosis	CPA	Sheath (Fr.)	Insertion method	Use fluoroscopy	Position (Zone)	Intervals for REBOA (min)	Hemodynam ics (SBP)	Complications	Definitive hemostatic control	Outcome
1	Low et al. [11]	1986	case series	1	NA	NA	mesenteric thrombosis	yes	11.5	cutdown	no	NA	NA	improved	NA	NA	Dead
2	Karkos et al. [12]	2001	case report	1	36	female	duodenum ulcer	no	NA	cutdown	no	I	30	70 → 140 mmHg	none	surgery	Alive
3	Hill et al. [13]	2010	case report	1	9	female	aortoesophageal fistula	yes	7	percutaneous	no	I	NA	improved	none	stent graft	Alive
4	Shigesato et al. [14]	2015	case report	1	79	female	duodenum ulcer	yes	9	percutaneous	no	I	150	40 → 240 mmHg	none	surgery	Alive

*CPA* cardiopulmonary arrest, *REBOA *resuscitative endovascular balloon occlusion of the aorta, *SBP* systolic blood pressure

In the present study, our trained acute care physicians could achieve REBOA procedures in the ED/ICU with a high degree of technical success. Blood pressure following REBOA did significantly increased compared with that before REBOA, and no significant differences detected between patients with UGIB in terms of almost all markers of end-organ dysfunction. A recent study reported a REBOA duration of >90 min in an animal model of hemorrhage-induced organ dysfunction, particularly that of the kidneys and liver. Our results were consistent with those reported by Markov et al. [9]. We also found clinically significant correlations between the total occlusion time of REBOA and lactate concentration and CRS measured at the beginning of resuscitation among patients with UGIB. Conventionally, these parameters are used to confirm a suspected massive hemorrhage and permit the earlier achievement of hemostasis or administration of massive transfusion if required [15]. Thus, it is very important to shorten the time from ED arrival to initiation of hemostatic procedures before hemodynamic collapse as much as possible. Although endoscopic treatment for UGIB is generally acceptable, it can be difficult to achieve complete hemostasis in some patients with a high lactate concentration and/or CRS. Excessive hemorrhage may rapidly become fatal and can be challenging to secure. In addition, it makes maintaining a visual field during endoscopy difficult. These complications result in significant time delays during procedures. In the present study, one patient (Patient No. 2) who had a transient response to initial resuscitation had his systolic blood pressure decrease to 80 mmHg. It was then decided to place an IABO catheter prior to endoscopic treatment, and an IABO catheter was promptly inserted at ED. His blood pressure subsequently increased following balloon inflation, and he underwent endoscopic treatment at ED. The bleeding vessel was visualized from the ulcer at the gastric angle, and the bleeding was arrested using balloon inflation. We believe that this case denotes the effectiveness of a balloon-assisted hemostatic technique in decreasing bleeding to secure a visual field and to reduce the volume of blood transfusion.

The immediately availability of an IABO catheter and the earlier introduction of REBOA have enabled us to sustain the circulation as an adjunct procedure until definitive hemostasis is attained. A formal algorithm that is utilized in a prospective manner is essential for UGIB treatment (endoscopy, AE, and surgery) and may shorten the occlusion time of REBOA. In the present study, one patient (Patient No. 5) with failed endoscopic hemostasis died within 24 h; the total occlusion time was 145 min. Although, REBOA for 60 min was reportedly to be well tolerated in an animal model of uncontrolled hemorrhagic shock, a recent report has suggested that partial REBOA could increase the survival time to 180 min while maintaining central blood pressure and carotid blood flow [10, 16]. In future, this method may be helpful for successful use of REBOA in patients with a high lactate concentration and a high CRS.

The major complications observed with REBOA included vessel injuries (i.e. aortic dissection, rapture and perforation), embolization, and peripheral ischemia. Recent reports have reported no vessel injuries caused by an IABO catheter or inflated balloon in trauma patients [17]. We routinely use ultrasonography to guide positioning of the IABO catheter during procedures and evaluate balloon placement using portable radiography after catheter deployment and there was no REBOA-related complications. Giuliani et al. support this result in the study showing that ultrasonography alone is safe and accurate as fluoroscopy for positioning and deployment of an IABO catheter [7].

However, this study has several limitations, particularly the small number of evaluated patients. Second, this was not a randomized, controlled trial because in the acute care setting, it is difficult to perform a randomized trial in a single emergency center. Furthermore, use of a propensity score is not suitable for a small sample size. Thus, large multi-institutional studies are essential for further evaluating the utility of REBOA against UGIB. Third, patients were allocated to REBOA use and treatments according to the decision of the attending lead acute care physicians, and this should be considered when interpreting our outcomes. Although patient selection was carefully performed, we believe the number of patients could have been greater. Consequently, our methods may be evaluated and incorporated into future studies to optimize the criteria for patient selection. Finally, application of this approach in other emergency centers may be limited by a lack of resources.

## Conclusion

Immediate availability of an IABO catheter device can perform REBOA in ER and ICU and achieve a high degree of technical success. Temporary aortic balloon occlusion is effective at improving hemodynamics and perfusion in patients with UGIB. Furthermore, the correlations of total occlusion time and high clinical Rockall score, as the most relevant one, lactate levels, and advanced age may be important for successful use of REBOA. Formal prospective study is warranted to clarify the role of this adjunct procedure in UGIB treatment.

## Abbreviations

AE: angio-embolization
APACHE: acute physiology and chronic health evaluation
AST: aminotransferases
BE: base excess
BUN: blood urea nitrogen
CK: creatine kinase
CRS: clinical rockall score
ED: emergency department
GBS: Glasgow-Blatchford bleeding score
IABO: intra-aortic balloon occlusion
LDH: lactate dehydrogenase
REBOA: resuscitative endovascular balloon occlusion of the aorta
SBP: systolic blood pressure
SI: shock index
UGIB: upper gastrointestinal bleeding
